# Os Biomarcadores Séricos Pré-Ablação podem ser Usados para Prever a Recorrência de Arritmia após Ablação de Fibrilação Atrial Guiada por Índice de Ablação?

**DOI:** 10.36660/abc.20240355

**Published:** 2024-07-23

**Authors:** Muhieddine Omar Chokr

**Affiliations:** 1 Universidade de São Paulo São Paulo SP Brasil Universidade de São Paulo, São Paulo, SP – Brasil; 2 Rede D'or de São Paulo São Paulo SP Brasil Rede D'or de São Paulo, São Paulo, SP – Brasil; 3 Faculdade de Medicina da Universidade de São Paulo Instituto do Coração São Paulo SP Brasil Instituto do Coração da Faculdade de Medicina da Universidade de São Paulo, São Paulo, SP – Brasil

**Keywords:** Biomarcadores, Fibrilação Atrial, Ablação por Cateter

O tratamento invasivo da fibrilação atrial (FA) tem se estabelecido como o mais eficaz no controle e cura dessa arritmia. Em sua forma paroxística, gatilhos provenientes do território das veias pulmonares são os responsáveis por deflagrar a FA na maioria dos casos, já na forma persistente, diferentes mecanismos eletrofisiológicos estão envolvidos, e quanto maior o tempo de evolução, mais complexa se torna a estratégia invasiva de tratamento. Apesar da evolução tecnológica com diferentes sistemas de mapeamento eletroanatômico, e com formação de lesões de radiofrequência na parede do átrio cada vez mais efetivas, as recorrências permanecem como um desafio a ser evitado.^1^

A criação de lesões de ablação duráveis durante o isolamento das veias pulmonares para FA é de importância crítica para prevenir a reconexão tardia das veias, que é responsável pela grande maioria das recorrências de arritmia em pacientes com FA paroxística. Apesar das melhorias na tecnologia, a proporção de veias que permanecem cronicamente isoladas após a ablação por radiofrequência continuou decepcionantemente baixa. Isto levou ao grande interesse na formação de lesões de ablação mais efetivas.^2^

Nos anos mais recentes o software Ablation Index (AI) (CARTO 3 V4, Biosense Webster, Inc., Diamond Bar, CA) é um novo marcador da qualidade da lesão que incorpora força de contato, tempo e potência em uma fórmula ponderada e demonstrou estimar com precisão a profundidade da lesão em estudos animais, além de se relacionar diretamente com a queda da impedância local.^3^ Diferentes trabalhos demonstraram menor taxa de reconexão quando se atinge o índice de 550 na parede anterior, e 400 na parede posterior do átrio esquerdo.^4^

Dessa forma, fica claro que variáveis intraprocedimento, como a característica da lesão e queda de impedância local podem predizer maior chance de recorrência. O grande desafio na pratica clínica, e conseguir predizer antes de realizar o procedimento, quais são os pacientes com maior chance de recorrência clínica após a ablação por cateter.

Nos últimos anos vários biomarcadores, de atividade inflamatória, hormonais, e de stress sobre a parede do miocárdio tem se relacionado a maior chance de recorrência da arritmia após a ablação.^5^ Ao encontro desses achados, nessa edição dos Arquivos Brasileiros de Cardiologia,^6^ os autores sugerem que embora isoladamente biomarcadores como TSH, Tiroxina livre (FT4) e BNP tenham baixo poder estatístico para predizer a recorrência, a medida que estão alterados a probabilidade de sobrevida livre de arritmia reduz progressivamente (87,1% se nenhum vs. 83,5% se um vs. 75,1% se dois vs. 43,3% se três biomarcadores estão elevados, p<0,001). Pacientes com três biomarcadores "anormais" têm três vezes maior probabilidade de recorrência, comparativamente com os que não têm nenhum biomarcador "anormal" (HR=2,88 [IC 95%, 1,39-5,17], p=0,003).

De forma interessante, a elevação nos biomarcadores foi suficiente para predizer recorrência após ablação da forma paroxística, mas não da forma persistente. Esses achados podem sugerir que o maior remodelamento anatômico e fibrose identificados na forma persistente, podem ter papel mais importante nas recorrências do que o estado inflamatório do paciente avaliados por meio da medida dos biomarcadores no presente estudo.^6^ Além disso, do ponto de vista de plausibilidade biológica, pode sugerir a utilização da avaliação dos biomarcadores em fases iniciais da doença, mas não em condições avançadas com remodelamento já estabelecido.

A proposta dos autores^6^ é interessante, e com certeza será uma variável adicional a ser utilizada pelos programas de inteligência artificial que tentam não só predizer os pacientes com maior chance de recorrência, mas que em um futuro próximo poderão identificar quais são os pacientes que necessitam de estratégias adicionais de ablação em adição ao isolamento das veias pulmonares na tentativa da manutenção do ritmo sinusal a longo prazo.
