# Supplementary material for: Os Biomarcadores Séricos Pré-Ablação podem ser Usados para Prever a Recorrência de Arritmia após Ablação de Fibrilação Atrial Guiada por Índice de Ablação?
Source: Arq Bras Cardiol. 2024 Jul 23;121(4):e20240355. [Article in Portuguese] doi: 10.36660/abc.20240355 (PMC11364445; doi:10.36660/abc.20240355)
Supplement: Translation

# Translation: Can Pre-Ablation Serum Biomarkers be Used to Predict Arrhythmia Recurrence after Ablation Index-Guided Atrial Fibrillation Ablation?

**DOI:** 10.36660/abc.20240355i

**Authors:** Muhieddine Omar Chokr

**Affiliations:** 1 São Paulo SP Brazil Universidade de São Paulo, São Paulo, SP – Brazil; 2 São Paulo SP Brazil Rede D'or de São Paulo, São Paulo, SP – Brazil; 3 São Paulo SP Brazil Instituto do Coração da Faculdade de Medicina da Universidade de São Paulo, São Paulo, SP – Brazil

**Keywords:** Biomarkers, Atrial Fibrillation, Catheter Ablation

Invasive treatment of atrial fibrillation (AF) has been established as the most effective treatment in controlling and curing this arrhythmia. In most cases, triggers originating from the pulmonary veins are responsible for triggering AF in its paroxysmal form. In its persistent form, different electrophysiological mechanisms are involved. The longer the evolution time, the more complex the invasive treatment strategy becomes. Despite the technological evolution with different electroanatomical mapping systems and the formation of increasingly effective radiofrequency lesions in the atrium wall, recurrences remain a challenge to be avoided.^1^

Creating long-lasting ablation lesions during pulmonary vein isolation for AF is of critical importance to prevent late vein reconnection. Such reconnection is responsible for the vast majority of arrhythmia recurrences in patients with paroxysmal AF. Despite technological improvements, the proportion of veins that remain chronically isolated after radiofrequency ablation is still disappointingly low. This has led to great interest in producing more effective ablation lesions.^2^

In more recent years, the Ablation Index (AI) (CARTO 3 V4, Biosense Webster, Inc., Diamond Bar, CA) software is a new lesion-quality marker that incorporates contact force, time, and power in a weighted formula. This index has been shown to accurately estimate lesion depth in animal studies, as well as having a direct relationship with the drop in local impedance.^3^ Different studies have shown a lower reconnection rate when indices of 550 and 400 are reached on the anterior and posterior walls of the left atrium.^4^

Thus, it is clear that intraprocedural variables, such as lesion characteristics and drop in local impedance, can predict a greater chance of recurrence. Predicting which patients have a greater chance of clinical recurrence after catheter ablation before performing the procedure is a major challenge in clinical practice.

In recent years, several biomarkers of inflammatory and hormonal activity, and stress in the myocardial wall have been related to a greater chance of arrhythmia recurrence after ablation.^5^ In this edition of the *Arquivos Brasileiros de Cardiologia*,^6^ the authors agree with these findings and suggest that biomarkers, such as TSH, free thyroxine (FT4), and BNP, have a low statistical power to predict recurrence, although isolatedly. As they are changed, the probability of arrhythmia-free survival progressively drops if none (87.1%), one (83.5%), two (75.1%), and three (43.3%) biomarkers are elevated (p<0.001). Patients with three "abnormal" biomarkers are three times more likely to have recurrence compared to those who do not have any "abnormal" biomarker (HR=2.88; 95%CI,=1.39-5.17; p=0.003).

Interestingly, elevation in biomarkers was sufficient to predict recurrence after ablation of the paroxysmal form, but not the persistent form. These findings suggest that a greater anatomical remodeling and fibrosis identified in the persistent form may have a more significant role in recurrences than the inflammatory status of patients assessed by measuring the biomarkers in the present study.^6^ From the point of view of biological plausibility, they may also suggest the use of biomarker assessment in the initial stages of the disease but not in advanced conditions with already established remodeling.

The authors’ proposal^6^ is interesting and will certainly be an additional variable to be used by artificial intelligence software. They try not only to predict patients with a greater chance of recurrence but which will also be able to identify patients who require additional ablation strategies soon, in addition to isolation of the pulmonary veins in an attempt to maintain sinus rhythm in the long term.
